# Epigenetic Modulation of Exercise Adaptation: The Role of Dietary Supplementation in Athletic Performance

**DOI:** 10.3390/genes17060618

**Published:** 2026-05-29

**Authors:** Agata Leońska-Duniec, Paulina Małkowska, Marek Sawczuk

**Affiliations:** 1 Faculty of Physical Education, Gdansk University of Physical Education and Sport, 80-336 Gdansk, Poland; agata.leonska-duniec@awf.gda.pl; 2 Department of Physiology in Health Sciences, Faculty of Health Sciences, Pomeranian Medical University, 71-210 Szczecin, Poland; paulina.malkowska@pum.edu.pl; 3 Institute of Physical Culture Sciences, University of Szczecin, 71-065 Szczecin, Poland

**Keywords:** epigenetics, exercise adaptation, nutritional supplementation, nutriepigenomics, DNA methylation, polyphenols, mitochondrial biogenesis, skeletal muscle

## Abstract

In high-performance sport, even small improvements in adaptation and recovery may influence competitive outcomes, increasing interest in molecular mechanisms that regulate training responses. Epigenetic processes represent a dynamic interface between exercise, nutrition, and long-term athletic adaptation. This narrative review summarizes current data on how dietary supplementation may modulate exercise-induced epigenetic remodeling and influence performance and recovery, focusing on mechanisms such as DNA methylation, histone modifications, and non-coding RNAs, as well as key nutrient groups with potential epigenetic activity, including polyphenols, omega-3 fatty acids, methyl donors, and selected vitamins. Evidence identified through targeted literature searches across major scientific databases indicates that several bioactive compounds can affect epigenetic pathways relevant to exercise adaptation. These mechanisms appear to modulate processes central to performance and recovery, including inflammation control, mitochondrial function, metabolic regulation, and tissue repair. Available evidence from multi-nutrient and combined exercise–nutrition interventions suggests potentially complementary effects on epigenetic regulation; however, human evidence remains limited and mechanistic interpretations should be considered preliminary. Overall, epigenetically informed supplementation offers a promising yet still developing approach and should be considered an adjunct to evidence-based training programs, balanced nutrition, and adequate recovery rather than a standalone performance-enhancing strategy.

## 1. Introduction

The pursuit of peak athletic performance has long been a quest for the perfect training regimen, the optimal diet, and the most effective recovery strategies. While traditional sports science focused on genetic potential and metabolic fuel, a new, more nuanced frontier has emerged: sports epigenetics. Epigenetics, literally meaning “above the gene”, refers to heritable changes in gene expression that occur without altering the underlying DNA sequence [1,2]. The concept of epigenetics was introduced by Conrad Waddington in 1939 together with the ‘epigenetic landscape’ framework describing how environmental and developmental factors shape phenotype expression without altering the DNA sequence. The three primary mechanisms of epigenetic control are DNA methylation, histone modification, and the action of non-coding RNAs (ncRNAs), particularly microRNAs (miRNAs) [3]. Beyond its classical role in developmental biology, epigenetics has become a critical framework in exercise physiology, offering mechanistic insight into how training stimuli are translated into long-term phenotypic adaptations. Unlike static genetic variants, epigenetic marks are dynamic, tissue-specific, and highly responsive to environmental inputs. This makes them uniquely suited to explain the variability in training outcomes observed among individuals exposed to similar exercise stimuli.

To provide a coherent conceptual bridge, it is important to first note that epigenetic regulation represents only one dimension of how individuals respond to dietary and environmental factors. While nutrigenetics examines how genetic variations influence the response to nutrients, nutriepigenetics investigates how dietary components can actively modify the epigenome. Thus, nutrigenetics, nutriepigenetics, and exercise epigenetics form a continuum, collectively explaining how inherited variants, environmentally induced chromatin changes, and nutrient-driven molecular regulation interact to shape training outcomes. In the context of sports science, this field offers a novel framework for understanding how specific supplements may fine-tune the molecular pathways involved in training adaptation and recovery [3,4]. One of the most promising applications of nutritional genomics lies in the field of sports performance. Genetic factors are estimated to account for roughly 50–80% of interindividual variation in lean body mass, with impacts detected on both ‘training-naive’ muscle mass and its growth response, highlighting the crucial role of genetics in determining physical performance and body composition [5].

**Figure 1:**
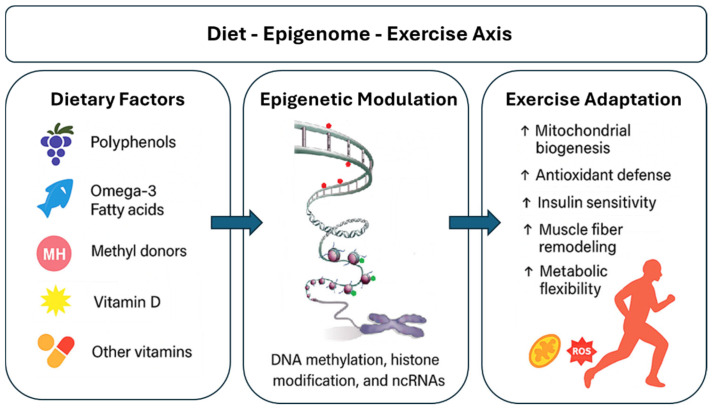
Dietary modulation of epigenetic mechanisms in exercise adaptation (↑—increase).

Exercise is a powerful environmental stimulus that acts as a potent epigenetic sculptor. It induces rapid and profound epigenetic remodeling in key tissues such as skeletal muscle, driving adaptation by altering the expression of genes related to mitochondrial biogenesis, muscle fiber type, and inflammation [6]. Because skeletal muscle is one of the most plastic tissues in the human body, it is particularly sensitive to epigenetic remodeling. Mechanical tension, metabolic stress, and endocrine signaling associated with exercise rapidly activate intracellular pathways that converge on chromatin-modifying enzymes. As a result, even acute exercise can induce measurable epigenetic shifts, which may accumulate over repeated sessions to support long-term phenotypic remodeling [7]. A single bout of exercise has been shown to trigger transient hypomethylation (a reduction in methylation levels) in the promoters of key metabolic genes such as *PPARGC1A* which encodes the peroxisome proliferator-activated receptor gamma coactivator 1-alpha (PGC-1α), resulting in increased transcriptional activity [8]. These epigenetic changes support enhanced mitochondrial biogenesis, improved glucose metabolism, and better oxidative capacity. Over longer-term interventions, such as endurance or combined training programs lasting several weeks or months, broader methylation changes occur across entire metabolic and signaling pathways, including those involved in insulin sensitivity, calcium signaling, and mitochondrial function [9,10]. Mechanistically, exercise-induced epigenetic remodeling is believed to arise from intracellular events linked to muscle contraction, such as calcium flux, altered AMP/ATP ratios, and the generation of reactive oxygen species, which affect the activity of methyltransferases and demethylases as well as the availability of methyl donors like S-adenosylmethionine (SAM). These biochemical shifts transiently reshape the epigenetic landscape, thereby modulating gene accessibility and transcriptional output [11]. Furthermore, growing evidence suggests that these epigenetic changes contribute to the so-called “muscle memory” phenomenon. Prior training can leave persistent epigenetic marks that enable a faster or more efficient adaptive response upon retraining [9,11]. In summary, exercise acts as an epigenetic architect, reprogramming skeletal muscle and other metabolic tissues through DNA methylation dynamics and related mechanisms (Figure 1).

These modifications fine-tune the expression of genes that govern mitochondrial biogenesis, inflammation, and energy metabolism, thereby forming a molecular bridge between physical activity and long-term health adaptation. Given the increasing interest in personalized training responses and precision nutrition, an up-to-date synthesis of how dietary compounds interact with exercise-induced epigenetic pathways is urgently needed. Despite rapid growth in the field, the current literature remains fragmented, with most studies focusing either on exercise epigenetics or nutritional epigenomics in isolation. This review addresses this gap by integrating these domains and highlighting nutriepigenetic strategies with potential practical relevance for athletes. Therefore, this review aims to explore novel research demonstrating that specific, targeted dietary supplements, often referred to as “nutriepigenetic” compounds, modulate exercise-induced epigenetic processes. By understanding this interplay, we can better appreciate the potential for these supplements to enhance and personalize the adaptive response to training.

The influence of environmental stimuli on epigenetic regulation extends beyond exercise alone and includes broader forms of environmental enrichment. Recent evidence suggests that enriched environmental conditions can modulate DNA methylation, histone modifications, and microRNA expression under stress-related conditions, supporting the concept that external lifestyle-related stimuli substantially contribute to epigenetic plasticity and adaptive responses [12]. Exercise may therefore be interpreted as one component of a wider environmental–epigenetic interaction network influencing physiological adaptation.

## 2. Exercise-Induced Epigenetic Regulation

The remarkable ability of skeletal muscle to adapt to training, a phenomenon often referred to as “muscle memory,” is fundamentally driven by epigenetic mechanisms. Exercise initiates complex signaling cascades that rapidly remodel the epigenetic landscape, ensuring that specific genes are activated at appropriate times to promote repair, growth, and metabolic efficiency [13]. Recent findings suggest that regular training is associated with a “younger” skeletal muscle methylome and coordinated transcriptomic shifts, reinforcing the notion that endurance and resistance exercise can slow epigenetic aging in muscle tissue [14].

Before delving into specific molecular pathways, it is important to clarify why the key genes discussed in this section matter for athletes. The products of genes such as *PPARGC1A*, *PDK4* (pyruvate dehydrogenase kinase 4, PDK4), *MEF2A* (myocyte enhancer factor 2A, MEFA2), or *PPARD* (peroxisome proliferator-activated receptor δ, PPAR δ) regulate mitochondrial biogenesis, fuel selection, oxidative capacity, and contraction-induced remodeling, core determinants of endurance, metabolic flexibility, and recovery. Their epigenetic regulation therefore directly shapes athletic performance by controlling the efficiency of energy production, substrate utilization, and training adaptation.

A central component of this adaptive process is the regulation of mitochondrial biogenesis, governed by a complex transcriptional network. A key regulator within this network is PGC-1α, which is often described as the “master regulator” of mitochondrial biogenesis. Exercise-induced hypomethylation at the *PPARGC1A* promoter represents a pivotal mechanism enhancing mitochondrial remodeling, and similar epigenetic shifts occur in other metabolic genes [15]. Downstream targets of PGC-1α, including *NRF1* (nuclear respiratory factor 1) and *TFAM* (mitochondrial transcription factor A) genes, are likewise epigenetically modulated to ensure coordinated mitochondrial proliferation and energy production. Direct human biopsy studies demonstrate that a single bout of exercise can remodel promoter methylation in key metabolic genes such as *PPARGC1A*, *PDK4*, and *PPARD* with acute hypomethylation closely paralleling increased transcriptional activity. The *PDK4* gene encodes an enzyme that inhibits the pyruvate dehydrogenase complex, shifting substrate utilization from carbohydrate oxidation toward lipid metabolism, which is a key adaptation during endurance exercise. Epigenetic studies show that exercise-induced hypomethylation of the *PDK4* promoter enhances its transcription, promoting metabolic flexibility and improved mitochondrial substrate selection [8]. Similarly, PPARδ is a nuclear receptor involved in fatty acid oxidation, muscle fiber-type switching, and mitochondrial function. Its expression increases after endurance exercise, accompanied by changes in DNA methylation and histone acetylation patterns that favor oxidative metabolism and resistance to fatigue [16].

In addition to *PPARGC1A*, several other genes are epigenetically modified in response to exercise, helping coordinate energy metabolism. The glucose transporter type 4 (*SLC2A4*; *GLUT4*) gene, which mediates insulin-dependent glucose uptake, exhibits reduced promoter methylation and enhanced expression after both acute and chronic exercise, leading to improved insulin sensitivity [10]. Likewise, carnitine palmitoyltransferase 1B (*CPT1B*), a rate-limiting enzyme in mitochondrial fatty acid transport, shows increased transcription following endurance training, reflecting enhanced oxidative capacity mediated by PPARδ activation [16,17]. Exercise also induces demethylation of *MEF2A*, which encodes a transcription factor involved in muscle fiber-type specification and glucose metabolism [10], as well as activation of *NFE2L2* which encodes a nuclear factor erythroid 2-related factor 2 (NRF2), which regulates antioxidant and mitochondrial gene expression. Moreover, contraction-induced stimuli can trigger locus-specific 5hmC modifications in exercise-responsive genes, adding a regulatory layer to rapid transcriptional activation [18].

**Figure 2:**
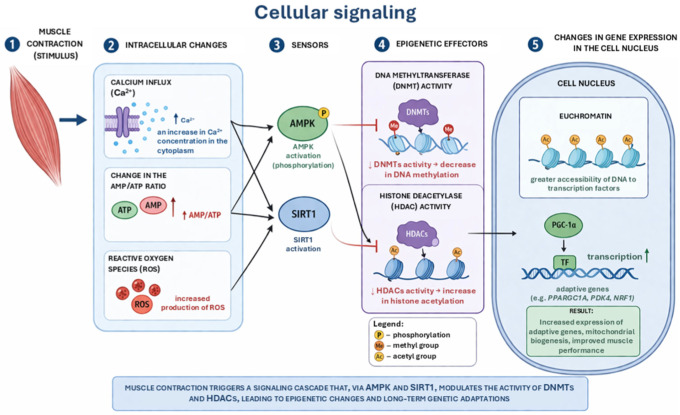
Intracellular signaling pathways linking muscle contraction to epigenetic remodeling. Exercise-induced increases in Ca^2+^ flux, ROS production, and the AMP/ATP ratio activate key metabolic sensors like AMPK and SIRT1. These sensors modulate the activity of epigenetic enzymes, including DNA methyltransferases (DNMTs) and histone deacetylases (HDACs), subsequently altering chromatin accessibility for pro-adaptive transcription factors (↑—increase; ↓—decrease; →—leads to; ⊢—inhibition).

Together, these gene-specific epigenetic responses form a coordinated molecular network that optimizes mitochondrial function, substrate utilization, and antioxidant defense in exercising muscle. In parallel, exercise also modulates central metabolic sensors that integrate cellular energy status with the epigenetic machinery. AMP-activated protein kinase (AMPK) acts as a primary energy sensor, activated by a decline in the ATP/AMP ratio during exercise. This activation leads to the phosphorylation of multiple targets, including PGC-1α, and indirectly affects the activity of epigenetic enzymes. Similarly, sirtuin 1 (SIRT1), a NAD^+^-dependent histone deacetylase, responds to elevated NAD^+^ availability and promotes transcription of adaptive genes such as *PPARGC1A* (Figure 2) [19]. A recent meta-analysis supports the consistent upregulation of SIRT1 following structured exercise interventions, aligning molecular changes with improved metabolic and functional adaptations [20].

Beyond energy metabolism, exercise-induced epigenetic reprogramming also governs muscle remodeling and growth. Training activates genes that regulate myogenesis, extracellular matrix organization, and hypertrophic signaling pathways. For instance, myogenic differentiation 1 (MYOD1) and myogenin (MYOG), which are master regulators of satellite cell activation and differentiation, show altered histone acetylation and methylation states following resistance exercise, reflecting enhanced chromatin accessibility at promoter regions [11]. Likewise, MEF2A, which controls both muscle fiber-type specification and structural remodeling, undergoes promoter demethylation after endurance exercise, promoting transcriptional activation [10]. Long-term training can also leave persistent epigenetic marks on genes involved in muscle growth and protein synthesis, such as *IGF1* (insulin-like growth factor 1, IGF-1), *MSTN* (myostatin), and *FNDC5* (fibronectin type III domain-containing 5), supporting the concept of an epigenetic “memory” of previous training [11].

Exercise also reshapes inflammatory and stress response pathways through epigenetic regulation, balancing pro- and anti-inflammatory signaling to promote tissue resilience. Moderate increases in reactive oxygen species (ROS) and cytokine release during exercise trigger transcriptional activation of protective genes rather than causing damage. For instance, *NFE2L2*, which encodes a master regulator of antioxidant defense, is epigenetically upregulated through histone acetylation and promoter demethylation, leading to enhanced expression of downstream targets such as *HMOX1* (heme oxygenase-1, HMOX1) and *SOD2* (superoxide dismutase 2, SOD2) [21]. In parallel, transient methylation changes have been reported in pro-inflammatory cytokine genes, including *IL6* (interleukin-6, IL-6) and *TNF* (tumor necrosis factor α, TNF-α), following acute exercise, reflecting the tightly controlled inflammatory signaling that facilitates recovery and adaptation [22]. These processes are further influenced by SIRT1 and AMPK activity, which suppress chronic inflammation by deacetylating nuclear factor kappa B (NF-κB) and enhancing antioxidant gene expression [19].

In addition to these epigenetic regulators, the methylenetetrahydrofolate reductase (MTHFR) gene (*MTHFR)* supports the maintenance of the methylation capacity required for exercise-induced epigenetic remodeling. MTHFR is a key enzyme in one-carbon metabolism, providing methyl groups necessary for DNA and histone methylation. Common polymorphisms, such as rs1801133, can reduce enzyme activity, altering methylation potential and affecting adaptive responses to training. Adequate folate and vitamin B_12_ availability is therefore essential to sustain optimal MTHFR activity, ensuring efficient epigenetic regulation of genes involved in energy metabolism, recovery, and muscle plasticity [23]. This genetic–epigenetic interplay suggests that an athlete’s *MTHFR* genotype may dictate their specific requirement for methyl donor supplementation to maintain the epigenetic flexibility needed for optimal training adaptation.

Beyond *MTHFR*, genetic variability in pathways related to antioxidant defense, inflammation, mitochondrial biogenesis, and nutrient metabolism may also influence exercise-induced epigenetic remodeling. Polymorphisms in genes such as *PPARGC1A*, *VDR*, *SOD2*, *TNF*, or fatty acid desaturase genes may partly determine individual responsiveness to nutritional interventions and adaptive training outcomes. These interactions further support the emerging concept of precision nutriepigenomics in sport.

Despite the rapid expansion of studies on exercise epigenetics and nutriepigenomics, no recent synthesis has focused specifically on how nutritional factors interact with exercise-induced epigenetic remodeling in athletes. Existing reviews typically address either molecular epigenetic mechanisms or sports nutrition in isolation. This creates a clear gap in the literature, which the present review aims to fill by integrating mechanistic, nutritional, and applied perspectives relevant to precision training.

**Figure 3:**
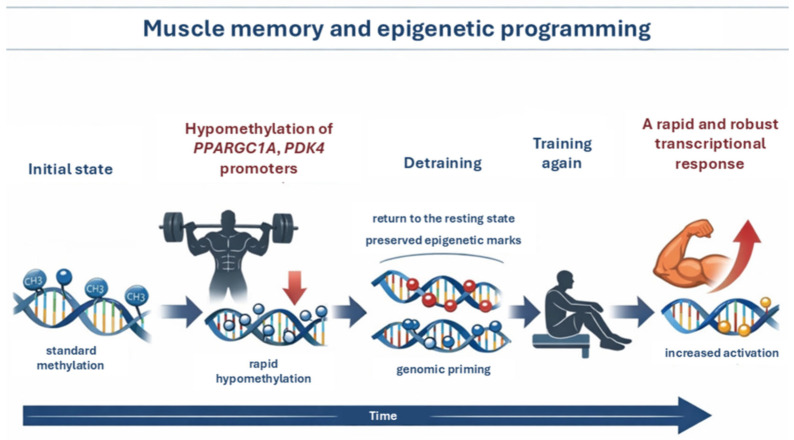
Epigenetic landscape of “muscle memory” and genomic priming. The initial training stimulus leads to acute hypomethylation of key metabolic genes (e.g., *PPARGC1A*, *PDK4*). While transcriptional levels return to baseline during detraining, specific epigenetic marks are retained (“epigenetic memory”), allowing for a more robust and rapid transcriptional response upon retraining (re-loading).

This integrated framework also provides the necessary context for understanding downstream phenomena such as epigenetic muscle memory and the potential of nutritional compounds to modulate long-term training adaptations. Persistent DNA hypomethylation of key myogenic and metabolic genes creates a state of ‘genomic priming,’ allowing the muscle to respond more robustly to future training stimuli even after long periods of detraining. Crucially, this window of epigenetic susceptibility defines a strategic opportunity where targeted dietary interventions, discussed in the following section, can act to further stabilize or enhance these molecular blueprints of adaptation (Figure 3).

## 3. Dietary Supplements as Epigenetic Modulators in Exercise Adaptation

Recent studies indicate that nutritional compounds with bioactive properties are increasingly recognized as modulators of epigenetic pathways. While epigenetics describes how training stimuli remodel gene regulation, nutrigenetics explains why individuals differ in these responses. Nutritional bioactives add a third dimension by modulating these same regulatory pathways. Integrating these perspectives is essential to understand how molecular mechanisms, genetic variability, and nutrient-derived signals jointly shape training outcomes. Among the major supplement classes with potential epigenetic effects are polyphenols, omega-3 fatty acids, methyl donors, and selected vitamins. However, the strength of evidence varies substantially across compounds: while some supplements have well-characterized epigenetic mechanisms, others rely on preliminary findings or indirect mechanistic inference. The concept of “epigenetic nutrition” has therefore attracted increasing interest as a theoretical bridge between molecular biology and applied sports science [24]. The most relevant dietary supplements and their primary epigenetic mechanisms are summarized in Table 1, illustrating how these compounds may act not only as nutritional aids but also as molecular signals that influence long-term physiological adaptation to exercise. However, it should be emphasized that much of the current evidence supporting nutriepigenetic interventions originates from in vitro studies, animal models, or short-term human protocols. Therefore, functional implications for athletes should be interpreted cautiously, and categorical statements about performance benefits have been replaced with more nuanced formulations. Longitudinal randomized controlled trials conducted specifically in elite athletic populations remain limited, making it difficult to determine the long-term functional relevance of many reported epigenetic modifications.

### 3.1. Polyphenols

Polyphenols are an extensive and chemically diverse family of plant secondary metabolites, all built around one or more phenolic rings and present in many staple foods such as fruits, vegetables, tea, cocoa, and coffee. They are usually grouped into flavonoids (flavanols, flavan-3-ols/catechins, anthocyanins, isoflavones, etc.) and non-flavonoids (phenolic acids, stilbenes, tannins, and others). Across these subclasses, polyphenols show broad biological activities; they scavenge reactive oxygen species, modulate antioxidant enzymes, and influence inflammatory signaling pathways, which underpin reported antioxidant, anti-inflammatory, antimicrobial, and cytoprotective effects in humans [70].

In sports contexts, interest in polyphenols stems from their ability to influence redox and inflammatory pathways and to modulate signaling networks linked to mitochondrial biogenesis, glucose and lipid metabolism, sometimes via epigenetic mechanisms affecting gene expression in muscle and other tissues [70,71]. Recent evidence shows that polyphenols can directly modulate epigenetic regulators, including DNMTs, histone modification enzymes, and non-coding RNAs, influencing chromatin accessibility and transcriptional programs relevant to metabolic adaptation. For example, EGCG has been shown to impact histone acetylation and methylation patterns, confirming the potential of dietary polyphenols to reshape the epigenome [72].

Physical exercise itself is a potent epigenetic modulator. Both acute and chronic bouts of exercise induce changes in DNA methylation, histone acetylation, and non-coding RNA expression in skeletal muscle, contributing to the transcriptional reprogramming underlying improvements in mitochondrial function, oxidative capacity, and glucose regulation. These exercise-induced epigenetic modifications have been demonstrated in humans, with studies documenting dynamic methylation changes in genes involved in energy metabolism and muscle adaptation [73,74]. Such findings highlight that training exerts direct effects on the epigenome that support long-term phenotypic adaptations.

Current “precision” or nutrigenomic approaches propose tailoring polyphenol type and dose to an individual’s genetic and epigenetic background and their capacity to manage exercise-induced oxidative stress, rather than applying uniform antioxidant strategies [70]. Because both nutritional polyphenols and physical exercise influence overlapping epigenetic targets there is growing interest in the potential synergy between supplementation and training. Polyphenols such as EGCG or curcumin may modulate DNMT activity or influence SIRT-dependent deacetylation, indicating a potential—but still insufficiently confirmed—interaction with potentially amplifying exercise-induced transcriptional responses [75,76].

Several studies have investigated the interaction between exercise and resveratrol on molecular pathways associated with mitochondrial adaptation and metabolic regulation. Resveratrol supplementation may prolong SIRT1- and AMPK-related signaling induced by exercise, although evidence for consistent functional benefits in athletes remains limited [20]. While some studies suggest that resveratrol may modulate SIRT1- and AMPK-related pathways in response to exercise, most findings derive from animal models or small human trials, and the functional relevance for athletes remains uncertain. Although direct evidence in humans on combined resveratrol supplementation and exercise-induced epigenetic remodeling is sparse, the known SIRT1-mediated deacetylation of histones and metabolic regulators suggests that resveratrol may amplify the epigenetic aspects of exercise adaptation. Experimental and clinical findings demonstrating exercise-induced hypomethylation and increased histone acetylation at promoters of metabolic genes support this shared mechanistic basis [73]. However, these hypotheses require clearer validation in well-controlled trials.

Furthermore, recent human trials indicate that dietary polyphenols can influence global and locus-specific DNA methylation markers, including those associated with biological aging trajectories. A randomized controlled study showed that polyphenol-rich dietary interventions modulate DNA methylation-based aging biomarkers, providing evidence that supplementation alone can affect systemic epigenetic programming [77].

### 3.2. Omega-3 Fatty Acids

Omega-3 fatty acids, particularly EPA and DHA from marine sources (fatty fish, fish oil, algal oil), are of growing interest in sports because of their effects on muscle, inflammation, and cell membrane function. In athletes, the omega-3 index (EPA+DHA as a % of erythrocyte fatty acids) is often low, typically around 3–5%, with less than 1% of athletes reaching the ≥8% level associated with cardioprotection, suggesting that many have room for improvement through diet or supplementation [78,79]. Human studies have shown that increasing dietary n-3 PUFA can modify DNA methylation in blood cells, particularly in genes related to immune and inflammatory pathways [80]. These epigenetic effects are currently considered context-dependent and vary between tissues, study designs, and baseline nutritional status.

From a sports perspective, several trials and reviews indicate that EPA+DHA supplementation can attenuate some aspects of exercise-induced muscle damage and soreness, likely via anti-inflammatory effects and altered membrane properties. Studies in resistance and eccentric exercise models report that weeks of EPA+DHA supplementation can reduce loss of strength, limitation of range of motion, and delayed onset muscle soreness after damaging exercise bouts; however, the overall evidence remains inconsistent and effect sizes are generally small and variable across protocols and populations [81,82]. Recent systematic and narrative reviews conclude that omega-3 supplementation may have a supportive role primarily as a recovery aid, supporting muscle regeneration, limiting chronic inflammation, and possibly preserving muscle mass during high training loads or disuse, rather than as a consistently demonstrated ergogenic intervention for acute performance enhancement [83,84,85]. However, these conclusions are largely based on short-term interventions, and long-term training adaptations have not been sufficiently studied.

Human nutriepigenetic studies have demonstrated that n-3 PUFA supplementation can alter methylation patterns in genes involved in immune regulation, inflammation, lipid metabolism, and mitochondrial pathways. For example, a randomized clinical trial reported fish oil supplementation to be associated with measurable, locus-specific DNA methylation changes in inflammatory and metabolic genes in peripheral blood [80]. Other studies suggest that EPA and DHA incorporation into cell membranes may influence the activity of epigenetic enzymes, including DNA methyltransferases and histone-modifying proteins, thereby modulating transcriptional responses to metabolic stress and inflammation [81]. Nevertheless, direct causal links between these molecular changes and functional performance outcomes in athletes remain unclear.

The interaction between exercise and omega-3 fatty acids also has epigenetic implications. Aerobic exercise induces acute hypomethylation in skeletal muscle metabolic genes and modifies methylation in circulating immune cells [73]. When combined with n-3 PUFA supplementation, these changes have been reported in some studies to be more pronounced, with endurance exercise combined with omega-3 supplementation associated with more favorable DNA methylation patterns in pathways related to inflammation and immune function compared to exercise alone [86]. This suggests that omega-3-induced anti-inflammatory effects may potentially complement exercise-triggered metabolic signaling; however, their magnitude, reproducibility, and long-term physiological relevance in athletic populations remain insufficiently established.

The combination of aerobic exercise and n-3 PUFA supplementation has been shown to induce more pronounced and favorable DNA methylation changes in peripheral blood cells than exercise alone. This suggests that the anti-inflammatory epigenetic effects of omega-3s complement the acute metabolic signaling of exercise, creating a more robust adaptive environment for recovery and tissue repair [87]. However, current evidence does not yet allow firm conclusions regarding clinical or performance-relevant outcomes of these epigenetic modifications.

### 3.3. Methyl Donors

Methyl donors are the next group of nutrients that feed one-carbon metabolism, generating S-adenosylmethionine (SAM), the universal methyl donor for DNA, RNA, and histone methylation. Key components include folate, choline, betaine, methionine, as well as B_2_, B_6_, and B_12_ vitamins, which act as cofactors in this network. By altering SAM and S-adenosylhomocysteine (SAH) levels, these nutrients can modulate DNA methyltransferase activity and thereby influence global and gene-specific DNA methylation [88,89]. This regulatory effect is well established at the biochemical level, whereas its magnitude in specific physiological contexts, such as exercise adaptation, appears to be variable.

Human data show that higher dietary methyl donor scores are associated with a more favorable metabolic profile and lower odds of metabolically unhealthy obesity. In contrast, specific methyl donor patterns have been linked to differences in body composition and cardiometabolic risk [90]. Other studies report that variation in methyl donor intake can modify DNA methylation of genes involved in growth, lipid metabolism, and obesity risk, such as *IGF2*, *LEP*, and other metabolic regulators [91,92]. However, these associations are primarily observational and do not establish causality between dietary intake and long-term epigenetic remodeling.

Emerging human and mechanistic research also shows that methyl donors interact with pathways relevant for exercise responses. Physical activity itself increases the turnover of one-carbon metabolites, elevates demand for folate-dependent nucleotide synthesis, and alters homocysteine metabolism, suggesting that athletes may have unique methyl donor requirements compared to sedentary individuals [93,94]. Moreover, exercise has been shown to acutely reduce circulating folate and B_12_ levels, indicating that intense or prolonged training may transiently lower methylation capacity [95]. These findings suggest a potential interaction between training load and methyl donor availability, although the functional consequences remain insufficiently defined. One-carbon metabolism and methyl donor status are also increasingly discussed as modifiable determinants of the magnitude and persistence of training-induced epigenetic remodeling in skeletal muscle and other tissues, although direct intervention data in athletes are still limited [96]. Recent experimental studies demonstrate that altering methyl donor intake can influence exercise-responsive loci. For example, folate or choline deficiency has been associated with altered DNA methylation responses to endurance-type exercise in metabolic and mitochondrial genes, whereas adequate methyl donor availability appears to support more stable exercise-responsive methylation patterns in genes involved in mitochondrial biogenesis and metabolic adaptation, including *PPARGC1A*, *NRF1*, and *TFAM* [13,97]. Importantly, these findings are derived mainly from animal and limited human studies, which restricts their direct translation to athletic populations.

In practical terms, this suggests that maintaining adequate, but not excessive, intakes of methyl donors may in the future be considered within precision nutrition frameworks aimed at supporting exercise-induced epigenetic responses. However, this remains a theoretical application rather than an evidence-based recommendation. Given that excessive intake of certain methyl donors, particularly folic acid or methionine, may be associated with adverse metabolic or cardiovascular effects, future guidelines will need to balance sufficiency with safety and consider inter-individual variability related to genetics, baseline nutritional status, and training load [98]. At present, there is insufficient evidence to define athlete-specific methyl donor intake recommendations based on epigenetic outcomes.

### 3.4. Vitamins

Other vitamins also interact with training adaptations and, indirectly, with epigenetic regulation. Vitamin D is the most intensively studied in athletes. Cross-sectional data indicate that a large proportion of athletes, especially in indoor and winter sports, have insufficient 25(OH)D levels, which is associated with reduced muscle strength, impaired power, and a higher risk of illness and injury [99,100,101]. However, these associations are influenced by multiple confounding factors, including training load, sun exposure, and overall nutritional status. Mechanistic and interventional studies indicate that vitamin D, via the specific receptor (VDR), modulates myoblast proliferation and differentiation, fiber size, mitochondrial function, and the expression of genes involved in muscle anabolism, oxidative stress defense, and recovery after damage [102,103]. Because VDR signaling involves chromatin remodeling and transcriptional regulation of extensive gene networks, vitamin D is increasingly recognized as an epigenetically active micronutrient capable of shaping training-induced gene expression, particularly in skeletal muscle and immune cells [100,104]. Nevertheless, the extent to which these molecular effects translate into meaningful performance adaptations in athletes remains incompletely understood.

Recent large-scale trials, such as the DO-HEALTH study, indicate that combining vitamin D supplementation with omega-3 fatty acids and structured exercise can produce additive effects on slowing biological aging, as measured by DNA methylation clocks, suggesting a multi-nutrient strategy can synergistically support epigenetic health [86,105]. However, the clinical relevance of these changes for athletic performance or recovery has not yet been established.

In contrast, high-dose supplementation with the classical antioxidant vitamins C and E has raised concerns in sports. While some trials show that short-term (3–7-day) high-dose vitamin C and E may reduce markers of acute muscle damage and hemolysis following very strenuous efforts or competition [106]. Several studies and reviews report that chronic these vitamins supplementation during training may attenuate exercise-induced increases in mitochondrial proteins, anabolic signaling, and hypertrophy, particularly in younger or resistance-trained individuals [107,108,109,110]. The likely explanation is that supraphysiological antioxidant intake dampens redox-sensitive signaling pathways (e.g., PGC-1α-mediated mitochondrial biogenesis, mTOR-related hypertrophic signaling) that help drive training adaptation. Because many of these pathways converge on transcription factors and chromatin-modifying enzymes, excessive antioxidant vitamin use is increasingly seen as potentially counterproductive from an epigenetic adaptation standpoint, whereas obtaining vitamins C and E from a diet rich in fruits, vegetables, and nuts is considered safer and sufficient for most athletes [107,111]. Importantly, these findings are not fully consistent across studies, and individual responses may vary depending on training status and dietary background.

Supplementation targeting epigenetic pathways should be integrated into broader strategies like training periodization, recovery, and overall dietary quality. While polyphenols, omega-3s, methyl donors, and vitamins each influence gene regulation through different biochemical mechanisms, their combined effects are hypothesized to interact at the level of shared signaling and transcriptional networks. However, current evidence supporting meaningful synergistic epigenetic effects in athletes remains limited and largely indirect. At present, such interactions should be considered speculative and context-dependent rather than established physiological principles. These effects may vary according to genetic background, training intensity, and baseline nutrient status, highlighting the need for individualized nutritional approaches rather than generalized supplementation protocols.

## 4. Integrated Nutrient–Exercise Epigenetic Crosstalk

The interaction between nutrients and exercise is best understood as a coordinated regulatory system rather than a collection of isolated molecular pathways. Exercise provides a strong physiological stimulus, while nutrients shape the cellular context in which this stimulus is interpreted. The epigenome integrates these signals, translating metabolic and environmental cues into changes in gene expression that support adaptation. In this framework, nutrients do not act as primary drivers of adaptation, but rather as modulators of the magnitude, direction, and temporal dynamics of exercise-induced molecular responses, including those mediated by DNA methylation, histone modifications, and non-coding RNAs [112].

### 4.1. Shared Metabolic–Epigenetic Nodes Linking Nutrients and Exercise

Endurance and resistance exercise acutely modify flux through one-carbon metabolism by altering methyl group turnover, homocysteine cycling, and methionine availability. These shifts influence the synthesis of SAM, the universal methyl donor required for DNA and histone methylation. A large body of evidence demonstrates that physical activity modulates DNA methylation patterns in skeletal muscle and blood cells, often in pathways relevant to energy metabolism and mitochondrial function [13]. Nutrients such as folate, vitamins B6/B12, choline, betaine, and methionine regulate the SAM:SAH ratio [113], meaning that suboptimal intake may reduce the methylation responsiveness to training stimuli.

Training acutely increases intramuscular acetyl-CoA levels, modulates mitochondrial NAD^+^ turnover, and elevates the activity of redox-sensitive transcriptional regulators. These shifts directly influence the activity of HATs (histone acetyltransferase) and sirtuin deacetylases. Evidence shows that acute and chronic exercise remodel histone acetylation states through NAD^+^-dependent sirtuin activation [114]. Nutrients such as vitamin D, omega-3 fatty acids, polyphenols, and creatine can alter mitochondrial redox state or acetyl-CoA flux, thereby intersecting with exercise-induced changes in chromatin accessibility. For example, vitamin D regulates chromatin remodeling through VDR–coactivator complexes and influences DNA methylation enzymes [115], omega-3 fatty acids modulate inflammatory gene methylation and enhance mitochondrial function [116], polyphenols may influence NAD^+^-dependent SIRT1 activity, promoting mitochondrial biogenesis and antioxidant signaling [117]. This metabolic convergence provides a plausible route through which supplementation modulates the kinetics of mitochondrial biogenesis, inflammatory resolution, or myogenesis.

### 4.2. Synergistic or Competing Effects of Supplementation and Training Stimuli

The interaction between nutritional supplementation and exercise produces a spectrum of synergistic, neutral, or competing epigenetic effects that extend beyond classical pathways of chromatin remodeling described earlier. These interactions arise because dietary compounds and physical training simultaneously influence hormonal signaling, mitochondrial stress responses, circadian regulation, and immunometabolic pathways, all of which converge on the epigenome [118].

A key level of interaction involves exercise-induced hormonal and cytokine responses (e.g., cortisol, catecholamines, IGF-1, IL-6), which can be modulated in sensitivity rather than directly overridden by nutritional factors [119]. Supplements that modulate endocrine activity, such as vitamin D, creatine, and omega-3 fatty acids, can alter the transcriptional sensitivity of target tissues to these hormones. For example, vitamin D enhances VDR binding to glucocorticoid-responsive elements [115], potentially shaping how exercise-induced cortisol surges influence chromatin accessibility. Similarly, creatine availability affects IGF-1-mediated anabolic signaling, which is partly governed by histone modifications and transcription factor recruitment in muscle fibers [120].

Nutrient–exercise interactions also manifest at the level of mitochondrial stress adaptation, which is intimately linked to epigenetic regulation via metabolites such as α-ketoglutarate, fumarate, and succinate—cofactors or inhibitors of dioxygenases regulating DNA and histone demethylation. Intense exercise transiently increases these intermediates, and supplementation with compounds like polyphenols or omega-3 fatty acids may modify their turnover or mitochondrial redox state, thereby indirectly influencing TET and JmjC enzyme activity [121]. This represents a context in which metabolic and epigenetic remodeling occur in parallel, although the degree of functional synergy in humans remains uncertain. An additional, but often overlooked, regulatory axis is circadian timing, which influences epigenetic enzyme activity and transcriptional responsiveness [122]. Training performed at different times of day modulates the expression of clock-controlled genes such as *BMAL1*, *PER2*, and *CRY1*, many of which regulate chromatin modifiers. Nutrients including caffeine, polyphenols, and vitamin D have chronobiological effects, shifting or stabilizing circadian epigenetic rhythms [123]. When combined with exercise timing, supplementation may modulate circadian alignment of molecular responses; however, evidence in athletic populations remains limited.

Interactions also occur within immunometabolic pathways, as exercise acutely activates immune cells and induces inflammation-driven chromatin remodeling. Supplements such as omega-3 fatty acids, probiotics, and curcumin can redirect immune cell metabolism toward less inflammatory phenotypes, influencing histone acetylation and DNA methylation in circulating monocytes and lymphocytes during recovery [124]. These effects may contribute to a more regulated inflammatory resolution phase, although excessive suppression of inflammation could potentially interfere with adaptive remodeling processes.

Finally, nutrient–exercise crosstalk extends to the regulation of transcription factor networks that orchestrate adaptation. Compounds such as polyphenols, caffeine, or nitrate can modify the activation thresholds of key transcription factors (PGC-1α, NF-κB, FOXO3, and HIF-1α) whose downstream genomic effects depend on chromatin accessibility shaped by exercise [125,126]. These effects are likely dose- and context-dependent, and their translation into meaningful performance outcomes remains incompletely characterized.

### 4.3. The Role of Non-Coding RNAs in Nutrient–Exercise–Epigenetic Integration

Non-coding RNAs (ncRNAs), including microRNAs (miRNAs), long non-coding RNAs (lncRNAs), and emerging circular RNAs (circRNAs), represent a regulatory interface through which metabolic signals from both nutrients and exercise are translated into durable epigenetic and transcriptional outcomes. Their importance stems from the fact that ncRNAs function not only as downstream markers of cellular stress or adaptation, but also as active orchestrators of chromatin remodeling, transcriptional coordination, and post-transcriptional regulation in skeletal muscle and immune tissues [15].

Exercise induces pronounced and time-dependent shifts in the expression of muscle-enriched miRNAs (myomiRs), including miR-1, miR-133a, and miR-206, which regulate satellite cell activation, mitochondrial biogenesis, angiogenesis, and hypertrophic responses. These dynamics have been consistently demonstrated in human and rodent training studies [127]. Importantly, exercise-regulated miRNAs are also secreted into the circulation via extracellular vesicles, where they can influence gene expression in distant metabolic tissues, illustrating a systemic ncRNA-mediated mode of exercise communication [128].

Nutritional compounds further shape this ncRNA landscape. Vitamin D regulates miRNA expression profiles involved in immune regulation and stress responses, including inflammation-linked miRs such as miR-146a, miR-150, and miR-155 in adipocytes and macrophages, suggesting a mechanism by which vitamin D modulates gene regulatory networks via miRNA dynamics rather than solely via direct chromatin binding [129,130,131]. Dietary omega-3 polyunsaturated fatty acids modulate the expression of inflammation-associated microRNAs and influence metabolic pathways linked to mitochondrial and lipid metabolism [132]. Polyphenols modulate the expression of microRNAs implicated in oxidative stress and metabolic regulation, impacting inflammatory and metabolic pathways linked to metabolic flexibility [133]. Conversely, methyl donors (folate, choline, betaine, B-vitamins) can modulate miRNA expression patterns indirectly via effects on DNA methylation and epigenetic regulation mechanisms linked to one-carbon metabolism [134].

Beyond miRNAs, long non-coding RNAs act as scaffolds, guides, or decoys for chromatin-modifying complexes and other regulatory proteins, thereby influencing transcriptional programs in skeletal muscle following physical training. Endurance and resistance exercise training have been shown to differentially regulate the expression of multiple lncRNAs in skeletal muscle, with potential roles in pathways related to mitochondrial adaptation, fiber-type specification, and mechanosensitive signaling [135]. Nutrient status may additionally influence these processes through nuclear receptor signaling (e.g., VDR, PPARs), although evidence linking this directly to training adaptation remains limited.

Circular RNAs (circRNAs), defined by their covalently closed, exonuclease-resistant structures with high stability, are increasingly recognized as regulators of gene expression and metabolic processes in skeletal muscle. These molecules can act as miRNA sponges, transcriptional modulators, or scaffolds for protein complexes, and emerging evidence indicates that exercise interventions modulate non-coding RNA expression, including specific circRNAs, with potential roles in mitochondrial function, metabolic regulation, and adaptive responses to endurance versus resistance training stimuli [136]. Although research integrating circRNAs with sports supplementation is still emerging, their molecular properties suggest they may function as persistent mediators of nutrient–exercise interactions.

Collectively, nutrient–exercise interactions represent a multilayered regulatory system in which metabolic, hormonal, and RNA-mediated mechanisms converge on epigenetic regulation. While this framework helps explain how supplementation may influence adaptation, it is important to emphasize that most evidence remains experimental or indirect. From the perspective of sports nutrition, epigenetic regulation currently serves more as an explanatory framework than a fully actionable intervention tool.

At present, the most substantiated role of supplementation (polyphenols, omega-3 fatty acids, methyl donors, and selected vitamins) is to modulate the physiological context of training rather than to directly reprogram epigenetic outcomes in a predictable manner. Further research is required before these mechanisms can be translated into evidence-based recommendations for athletes.

## 5. Practical Applications and Ethical Considerations

The practical application of epigenetic modulation through dietary supplementation in sports represents an innovative intersection of molecular nutrition, performance physiology, and personalized medicine. Epigenetic regulation is a dynamic process responsive to physiological stimuli, and physical exercise itself constitutes one of the strongest modulators of epigenetic marks in skeletal muscle and other tissues, driving adaptations relevant to energy metabolism, mitochondrial biogenesis, and structural remodeling [137]. These interventions are designed to fine-tune, rather than replace, the body’s natural adaptive mechanisms by influencing epigenetic marks. Regular physical exercise itself is one of the strongest epigenetic stimuli, but targeted supplementation may augment specific pathways of adaptation. In this context, targeted nutritional strategies are best viewed as tools to bias or support distinct cascades, such as mitochondrial biogenesis, inflammation resolution, or hypertrophic signaling [137].

From an applied standpoint, supplementation should be individualized and periodized according to the athlete’s training cycle, baseline nutrient status, and, where available, relevant genetic or epigenetic information. The field of sport nutrigenomics emphasizes that inter-individual differences in genes related to nutrient metabolism, inflammation, and antioxidant defense can influence both performance outcomes and responses to dietary strategies [138]. In practice, this means that the same supplement (for example, omega-3 fatty acids or vitamin D) may have minimal additional effect in a well-nourished athlete, but a measurable molecular and functional impact in an athlete with a clear deficiency or suboptimal status [85,139,140].

Effective implementation of epigenetically informed supplementation in sport requires systematic monitoring of relevant biomarkers rather than empirical dosing. Biomarker assessment, such as serum 25(OH)D for vitamin D status, an erythrocyte omega-3 index for n-3 PUFA, and selected markers of inflammation or one-carbon metabolism (e.g., homocysteine, folate, vitamin B12), helps to identify athletes who are likely to benefit most from supplementation, tailor doses and timing to the individual, and avoid chronic over-supplementation with little added benefit. This biomarker-guided approach is consistent with broader efforts to link nutrition, exercise, and biological aging using epigenetic clocks, as seen in recent trials combining vitamin D, omega-3, and structured exercise in older adults to assess effects on epigenetic age [86]. Additionally, the optimal dose for epigenetic effects often exceeds standard dietary recommendations, and the duration of supplementation must be sufficient to stabilize epigenetic modifications, which may require sustained intake over weeks or months. The timing of supplement intake relative to training can influence adaptive responses. For instance, post-exercise ingestion of anti-inflammatory bioactives such as omega-3 fatty acids or polyphenols may favorably modulate post-exercise inflammatory and epigenetic signaling, whereas continuous provision of methyl donors supports maintenance of methylation capacity in tissues undergoing repeated rounds of transcriptional regulation and repair.

It is important to acknowledge that most of the current evidence on diet–epigenome interactions comes from clinical or general populations rather than from elite sport. While these studies are not conducted in high-performance athletes, they illustrate the methodological framework for integrating molecular markers into personalized interventions. Reviews of nutriepigenetics and sport nutrigenomics emphasize that, although mechanistic and early clinical data are promising, robust randomized controlled trials directly linking specific epigenetic changes to meaningful performance or recovery outcomes in athletes are still scarce [137]. Furthermore, many available studies assess surrogate molecular endpoints rather than direct sport-specific performance outcomes, which limits the immediate translation of mechanistic findings into evidence-based athletic practice. In addition, the available literature is characterized by substantial heterogeneity in study design, supplementation protocols, participant training status, epigenetic endpoints, and intervention duration, which complicates direct comparisons between studies and limits the generalizability of findings. As a result, the practical application of epigenetically targeted supplementation should be conservative and context-dependent. These strategies are best used as adjuncts layered onto well-structured training, adequate energy availability, high-quality diet, and sufficient sleep, rather than as stand-alone performance enhancers.

The ethical landscape surrounding epigenetic supplementation in sport is nuanced. While the consumption of nutrients for health and performance is well established, the deliberate manipulation of gene expression, even though natural dietary compounds, raises questions about fairness, long-term safety, and the potential erosion of sporting integrity [141,142]. If such interventions provide molecular advantages that persist beyond the supplementation period, they may blur the line between permissible enhancement and genetic modification. Informed consent and athlete education are essential ethical safeguards. Practitioners must clearly communicate the experimental nature of epigenetic interventions and avoid overstating benefits or minimizing uncertainties [143,144]. Athletes should be empowered to make informed decisions about supplement use, supported by transparent data and independent scientific oversight. Furthermore, equity of access remains a pressing concern. Advanced nutriepigenetic formulations or personalized interventions may be costly, creating disparities in access and potentially widening the performance gap between elite and resource-limited athletes. Ensuring fair distribution and open access to knowledge is a matter of both sporting justice and global health ethics [145,146].

Ultimately, the responsible integration of epigenetic nutrition in sports must balance innovation with integrity. Interventions should prioritize athlete well-being, fairness, and transparency, aligning with the ethical principles outlined by international sporting bodies. Enhancement should never compromise safety or equity, and ongoing interdisciplinary research will be essential to clarify not only the scientific efficacy but also the moral legitimacy of epigenetic supplementation in human performance [141,145].

## 6. Future Directions

The next decade is likely to gradually expand our understanding of how epigenetic information is used in sport, shifting the concept of an “epigenetic edge” from theoretical frameworks toward more cautiously interpreted applied research contexts. Large-scale epigenome-wide association studies (EWAS) in athletes are expected to play an important exploratory role, although their practical translation remains limited. By scanning hundreds of thousands of CpG sites across the genome, EWAS can identify methylation patterns associated with specific training phenotypes (e.g., endurance vs. strength specialists), exercise responsiveness, and susceptibility to overuse injury or illness. Early work already shows that elite endurance- and strength-trained athletes display distinct DNA methylation profiles in exercise-responsive genes compared with untrained controls, and that methylation at key regulatory loci correlates with physiological traits such as VO_2_peak [147]. Recent EWAS-scale analyses in elite soccer players further demonstrate that blood DNA methylation signatures can discriminate between athletes with higher versus lower non-contact injury risk, and link epigenetic age acceleration to widespread hypomethylation patterns [148]. Such approaches foreshadow potential future biomarker development for monitoring training adaptation, although clinical or practical implementation in sport is still premature.

A parallel avenue will be the refinement of epigenetic clocks and age-related biomarkers in high-performance sport. Epigenetic age estimators based on DNA methylation already reveal that elite athletes can differ from the general population in biological age, and that training load and injury history may leave detectable marks on methylation-derived age acceleration. Longitudinal studies that repeatedly measure epigenetic clocks over seasons, alongside performance metrics and injury surveillance, could in the long term potentially support their use for monitoring cumulative stress, recovery sufficiency, and long-term health trajectories in professional sport [149,150]. However, their current utility in day-to-day training decision-making remains unproven.

Advances in sequencing technology will further deepen mechanistic insight. Long-read sequencing and, especially, single-cell and single-nucleus approaches are already transforming our understanding of exercise adaptation at the level of individual cell types within muscle and other tissues. Single-cell RNA sequencing in human skeletal muscle has shown that acute and chronic exercise remodel the composition and transcriptional state of multiple cell populations, including myofibers, satellite cells, endothelial cells, and fibro-adipogenic progenitors [151]. Integrated single-cell multiome studies that combine transcriptomics with chromatin accessibility in the same nucleus now map how endurance exercise rewires gene regulatory circuits in specific muscle cell types. Extending these designs to single-cell methylomics will allow precise localization of DNA methylation changes to distinct fiber types or stem cell niches [152,153] although direct evidence linking such cell-specific epigenetic differences to athletic performance outcomes is still lacking.

Beyond individual technologies, future research will increasingly move toward multi-omics integration. Flagship projects such as the MoTrPAC endurance-training consortium have demonstrated how combining transcriptomics, proteomics, metabolomics, and epigenomic readouts across multiple tissues can generate a whole-organism map of training adaptations [154]. In sports science, emerging concepts such as “enduromics” and “resistomics” explicitly frame endurance- and resistance-training responses as multi-layered biological processes. However, these frameworks remain primarily research-oriented and require further validation before influencing applied sport practice. Importantly, when multi-omic approaches include nutritional and supplementation-related data, they may eventually help to better contextualize individual variability in response to training and diet, although such predictive applications remain speculative [152,155,156].

For epigenetic nutrition specifically, the most promising future direction lies in its integration into precision nutrition frameworks. Sport nutrigenomics already highlights how genetic variability in nutrient metabolism, inflammatory pathways, and antioxidant defenses modulates responses to diet and supplements; adding longitudinal epigenetic measures will help determine whether particular dietary patterns or compounds induce durable, beneficial shifts in gene regulation [138,156]. In this context, supplements with proposed epigenetic activity remain the most immediately relevant and practically investigated interface between nutrition and exercise-induced epigenetic adaptation.

An important challenge for future research will be the standardization of study design, training protocols, supplementation strategies, and epigenetic outcome measures. The heterogeneity of current methodologies complicates comparisons across studies and limits the development of clear practical recommendations for athletes.

Taken together, these developments suggest that the “epigenetic edge” in sport will increasingly be defined not by single nutrients or isolated biomarkers, but by integrated, multi-layered models of adaptation that combine EWAS data, single-cell and multi-omic technologies, and nutrigenomic profiling within a rigorous, ethically grounded framework. At present, however, most proposed nutriepigenetic applications remain mechanistically interesting but insufficiently validated for implementation in athletic practice, particularly in relation to supplementation strategies.

## Figures and Tables

**Table 1:** Dietary supplements acting as epigenetic modulators in the sport context.

Supplement	Main Natural Sources	Average Daily Dose	Epigenetic Mechanism	Potential Effect on Performance	Key References
**Polyphenols**					
Resveratrol	Grapes and red wine	100–500 mg	Modulation of sirtuins (SIRT1) and HDAC activity; suggested indirect influence on DNA methylation pathways	May improve muscle fatigue resistance and metabolic efficiency; evidence for direct ergogenic effects remains limited	[25,26]
Curcumin	Turmeric	80–200 mg	DNMT inhibition and histone acetylation modulation (preclinical evidence)	May reduce exercise-induced soreness and inflammation; human performance effects remain inconsistent	[27,28,29,30]
Quercetin	Various fruits and vegetables	200–1000 mg	Modulation of oxidative stress gene expression; possible indirect epigenetic regulation via signaling pathways	Mixed evidence for performance improvement; may support recovery and reduce perceived fatigue in some populations	[31,32,33]
Cocoa flavanols	Cocoas, chocolates, teas, red wines, fruits, cereals, beans, spices, and nuts	200–500 mg	Associated with changes in DNA methylation patterns in blood cells (limited human evidence)	May improve endothelial function and vascular responses to exercise; performance effects not consistent	[34,35]
Blueberry	Berries	75–150 g	May influence DNA methylation and histone modifications (preliminary evidence)	May support recovery and vascular function after exercise	[36,37]
EGCG	Green tea	250–1000 mg	Inhibition of DNMTs and HDACs (mainly experimental evidence)	May reduce oxidative stress and muscle damage; ergogenic effects remain inconclusive	[38,39]
Pycnogenol^®^	French maritime pine bark	100–800 mg	Possible modulation of DNA methylation and histone acetylation (limited evidence)	May improve exercise tolerance and recovery; evidence base still limited	[40,41]
Montmorency cherry juice	Montmorency cherries, also known as sour cherries	30 mL	Potential modulation of epigenetic regulation via antioxidant signaling (indirect evidence)	May enhance recovery and reduce post-exercise soreness	[42,43,44]
Ecklonia cava	Brown alga—Ecklonia cava	40 mg	Suggested regulation via AMPK/SIRT1 pathways with potential epigenetic interaction (preclinical)	May improve metabolic efficiency and reduce lactate accumulation	[45,46,47]
**Omega-3 fatty acids**					
EPA & DHA	Fatty fish, other seafood, and algae	3–4.4 g	Modulation of DNA methylation in inflammatory pathways; histone modifications (context-dependent evidence)	May reduce inflammation and support recovery; performance benefits are variable	[48,49,50]
**Methyl Donor**					
Folate	Leafy greens, legumes, cruciferous vegetables, and whole grains	400–800 µg	Central role in SAM cycle supporting DNA and histone methylation	Supports metabolic stability and recovery; effects on performance indirect	[51,52,53]
Betaine	Spinach, wheat products, and beets	2.5–5 g	Homocysteine remethylation and SAM availability support	May support methylation balance and muscle function; evidence in athletes limited	[54,55,56]
Choline	Eggs, red meat, poultry, fish, milk, and some vegetables	125–550 mg	Precursor of SAM; supports one-carbon metabolism and methylation capacity	May support neuromuscular function and recovery; performance effects unclear	[57,58,59]
Vitamins B2, B6, and B12	Meat, fish, liver, eggs, dairy, leafy greens, legumes, nuts, and seeds	B2 1.3–1.6 mg; B6 2 mg; B12 500 µg	Cofactors in one-carbon metabolism supporting methylation reactions	Support metabolic function and recovery; deficiency-dependent effects	[60,61]
Methionine	Meat, fish, eggs, dairy, nuts, beans, and whole grains	1.3–3 g	Methyl donor in SAM cycle supporting DNA/histone methylation	Supports metabolic and antioxidant balance; excessive intake may be unfavorable	[62,63]
**Other vitamins**					
Vitamin D	Sunlight exposure and fatty fish, red meat, eggs	1000–4000 IU	VDR-mediated chromatin remodeling and transcriptional regulation	Supports muscle and immune function; ergogenic effects inconsistent	[64,65,66]
Vitamins C+E	Vitamin C: fruit and vegetables; Vitamin E: seeds, nuts, oils, fish, and vegetables	500–1000 mg	No direct epigenetic effects on DNA methylation/histone modification confirmed	May reduce oxidative stress acutely but may blunt training adaptations when chronically supplemented	[67,68,69]

## Data Availability

No new data were created or analyzed in this study.

## Abbreviations

The following abbreviations are used in this manuscript:

DNA	deoxyribonucleic acid
RNA	ribonucleic acid
ncRNA	non-coding RNAs
miRNA	micro-RNA
*PPARGC1A*	peroxisome proliferator-activated receptor gamma coactivator 1-alpha gene
PGC-1α	peroxisome proliferator-activated receptor gamma coactivator 1-alpha
AMP	adenosine monophosphate
ATP	adenosine triphosphate
SAM	S-adenosylmethionine
NAD	nicotinamide adenine dinucleotide
*NRF1*	nuclear respiratory factor 1 gene
*TFAM*	mitochondrial transcription factor A gene
*PDK4*	pyruvate dehydrogenase kinase 4 gene
PDK4	pyruvate dehydrogenase kinase 4
*PPARD*	peroxisome proliferator-activated receptor δ gene
PPARδ	peroxisome proliferator-activated receptor delta
*SLC2A4*; *GLUT4*	glucose transporter type 4 gene
CPT1B	carnitine palmitoyltransferase 1B
*MEF2A*	myocyte enhancer factor 2A gene
MEF2A	myocyte enhancer factor 2A
*NFE2L2*	nuclear factor erythroid 2-related factor 2 gene
NRF2	nuclear factor erythroid 2-related factor 2
5hmC	5-hydroxymethylcytosine
AMPK	AMP-activated protein kinase
SIRT1	sirtuin 1
MYOD1	myogenic differentiation 1
MYOG	myogenin
DNMTs	methyltransferases
HDACs	histone deacetylases
ROS	reactive oxygen species
*IGF1*	insulin-like growth factor 1 gene
IGF1	insulin-like growth factor 1
MSTN	myostatin
FNDC5	fibronectin type III do-main-containing 5
HMOX1	heme oxygenase-1
SOD2	superoxide dismutase 2
*IL6*	interleukin-6 gene
IL-6	interleukin-6
*TNF*	tumor necrosis factor α gene
TNF-α	tumor necrosis factor α
NF-κB	nuclear factor kappa B
*MTHFR*	methylenetetrahydrofolate reductase gene
MTHFR	methylenetetrahydrofolate reductase
EGCG	epigallocatechin gallate
EPA	eicosapentaenoic acid
DHA	docosahexaenoic acid
VO_2_max	maximal oxygen uptake
n-3 PUFA	omega-3 polyunsaturated fatty acids
SAH	S-adenosylhomocysteine
*IGF2*	insulin-like growth factor 2 gene
*LEP*	leptin gene
25(OH)D	25-hydroxyvitamin D
VDR	vitamin D receptor
DO-HEALTH study	Vitamin D3–Omega-3–Home Exercise–Healthy Ageing and Longevity Trial
mTOR	mechanistic target of rapamycin
HAT	histone acetyltransferase
TET	ten-eleven translocation enzyme
JmjC	Jumonji C domain-containing enzyme
*BMAL1*	brain and muscle ARNT-like 1 gene
*PER2*	period circadian regulator 2 gene
*CRY1*	cryptochrome circadian regulator 1 gene
FOXO3	forkhead box O3
HIF-1α	hypoxia-inducible factor 1-alpha
lncRNAs	long non-coding RNAs
circRNAs	circular RNAs
myomiRs	muscle-enriched miRNAs
EWAS	epigenome-wide association studies
CpG	cytosine-phosphate-guanine
VO_2_peak	peak oxygen uptake
MoTrPAC	Molecular Transducers of Physical Activity Consortium
